# Newborn pulse oximetry screening for critical congenital heart defects

**DOI:** 10.1186/s12887-021-02520-7

**Published:** 2021-09-08

**Authors:** Sophie Jullien

**Affiliations:** Barcelona Institute for Global Health, University of Barcelona, Barcelona, Spain

**Keywords:** Screening, Congenital heart defects, Pulse oximetry, Newborn

## Abstract

We looked at existing recommendations and supporting evidence addressing the effectiveness of pulse oximetry effective for detecting critical congenital heart defects (CCHDs) in newborns. We also looked at the impact of timing of oximetry and the site of testing in the accuracy of screening, and at the potential harms and limitations of pulse oximetry screening,

We conducted a literature search up to the 13^th^ of August 2019 by using key terms and manual search in selected sources. We summarized the recommendations and the strength of the recommendation when and as reported by the authors. We summarized the main findings of systematic reviews with the certainty of the evidence as reported.

Current evidence supports consistent accuracy for detection of CCHDs in newborns by pulse oximetry screening in addition to antenatal ultrasonography and clinical examination. Overall, early diagnosis of CCHD with pulse oximetry is judged to be beneficial and cost-effective, and potential harms associated with false-positive tests are not serious, while missing CCHDs and other serious diseases detected by hypoxaemia in absence of pulse oximetry screening can lead to serious consequences. The site of testing (post-ductal versus pre- and post-ductal) had no significant effect on sensitivity nor specificity for detection of CCHDs.

## Background

### Introduction

The World Health Organization (WHO) European Region is developing a new pocket book for primary health care for children and adolescents in Europe. This article is part of a series of reviews, which aim to summarize the existing recommendations and the most recent evidence on preventive interventions applied to children under five years of age to inform the WHO editorial group to make recommendations for health promotion in primary health care. In this article, we looked at existing recommendations and supporting evidence addressing the effectiveness of pulse oximetry effective for detecting critical congenital heart defects (CCHDs) in newborns. We also looked at the impact of timing of oximetry and the site of testing (post-ductal versus pre- and post-ductal) in the accuracy of screening, and at the potential harms and limitations of pulse oximetry screening.

### Why is screening for critical congenital heart defects important?

CCHD is defined as any cardiac lesion from which infants die or require surgery or cardiac catheterization within the first 28 days of life to prevent death or severe end-organ damage [1, 2]. Early detection of CCHD before acute cardiovascular collapse leads to improved cardiopulmonary and neurological outcomes [3]. However, most newborns are asymptomatic at birth. Newborn screening for CCHD can help identifying some cases to allow prompt diagnosis and treatment, and may prevent disability or fatal outcome [4].

### Context

Congenital heart defects (CHDs) constitute the most common group of birth defects, with a prevalence of around 6 to 11 per 1000 live births for moderate and severe cases [2, 5–8]. They account for up to 10% of all infant deaths, and 46% of deaths related to congenital malformations [5, 9]. About 25% of CHDs are life-threatening CCHDs. Antenatal ultrasound screening and newborn clinical examination are already established methods to detect malformations such as CCHDs. However, it was estimated that antenatal ultrasound can detect around two thirds of CCHDs (sensitivity of 68.1%; 95% confidence interval [CI] 59.6 to 75.5%), and that the newborn examination also has a low detection rate [5, 6]. Indeed, detection of hypoxaemia by visual assessment of the newborn colour has limitations, and cardiac murmurs are not always present in cases of CCHDs and accidental murmurs can be heard in up to 60% of healthy newborns [6]. The combination of antenatal ultrasonography and clinical examination of the newborn lead to sending home up to 30% of cases of CCHDs before diagnosis, with mortality rates up to 50% [5, 6]. Several studies have reported that adding routine pulse oximetry (PO) to the antenatal ultrasound screening and clinical examination of the newborn can potentially improve the detection of CCHDs [10]. PO is a simple, non-invasive and painless tool that measures oxygen saturation, and therefore could detect CCHDs with ductal-dependent systemic or pulmonary blood flow that usually present with hypoxemia. Seven severe lesions have been identified as primary targets for screening by PO; those are hypoplastic left heart syndrome, pulmonary atresia, tetralogy of Fallot, total anomalous pulmonary venous return, transposition of the great arteries, tricuspid atresia, and truncus arteriosus [11].

## Key questions

1. Is PO effective for detection of CCHDs in newborns?
2. Does timing of oximetry have an impact in the accuracy of screening?
3. Is pre- and post-ductal more effective than post-ductal measurement for detection of CCHDs?
4. What are the harms and limitations of PO screening?

Cost-effectiveness of the implementation of PO screening and acceptability to parents and health workers are beyond the scope of this summary but were assessed by others [6, 9, 11, 12].

## Search methods and selected manuscripts

We described the search methods, data collection and data synthesis in the second paper of this supplement (Jullien S, Huss G, Weige R. Supporting recommendations for childhood preventive interventions for primary health care: elaboration of evidence synthesis and lessons learnt. BMC Pediatr. 2021 10.1186/s12887-021-02638-8).

The search was conducted on the 13^th^ of August 2019, by manual search and by using the search term “congenital heart” OR “congenital cardi*”. We did not find any recommendations from the WHO. No document was identified from the US Preventive Services Task Force (USPSTF) (published recommendations or recommendations in progress) or the PrevInfad workgroup (Spanish Association of Primary Care Pediatrics), but we did include the consensus document with recommendations from the Spanish National Neonatal Society, Spanish Association of Paediatrics (AEP, from acronym in Spanish). We did not find recommendations from the National Institute for Health and Care Excellence (NICE) other than the physical examination of the newborn that should include ‘heart; check position, heart rate, rhythm and sounds, murmurs and femoral pulse volume’ [13]. From the Centers of Disease Control and Prevention (CDC) website, we included a fact sheet and recommendations addressing this topic. We also included the 2011 recommendations from the American Academy of Pediatrics (AAP) and their comprehensive website within the “Program to Enhance the Health & Development of Infants and Childre”’. The Royal College of Paediatrics and Child Health (RCPCH) included this topic within their chapter on “Physical examination.” Finally, we also included the recommendations stated by the UK National Screening Committee (NSC) in their recommendations report from 2014-15, and their current update on this matter.

The search in the Cochrane library by using the search strategy ‘congenital heart OR congenital cardi*’ in titles, abstracts or keywords returned 41 reviews and three protocols. By screening the titles and abstracts, we included one systematic review and two protocols. However, one protocol entitled ‘Routine screening by echocardiography to reduce morbidity and mortality from congenital heart disease in neonates with Down syndrome’ dated from 2005, and the other one, entitled ‘Clinical assessment for diagnosing congenital heart disease in newborn infants with Down syndrome’ was out of date and consequently withdrawn from the authors and editors of Cochrane Neonatal. Therefore, we did not contact authors from any of these two protocols and only included one Cochrane review published in 2018. By looking at references from included manuscripts and by hand search, we identified two documents endorsed by the AAP for inclusion (one published by Kemper et al in 2011, and one published by Martin et al in 2013) and one report from a pilot study conducted in the UK (published by Evans et al in 2016).

All the included manuscripts for revision in this article are displayed in Table 1.

**Table 1:** Included manuscripts for revision

**Sources**	**Final selected manuscripts**
**WHO**	None
**USPSTF**	None
**PrevInfad**	None
**AEP**	• The Spanish National Neonatal Society Recommendations [6]
**CDC**	• Fact sheet and recommendations [4]
**NICE**	None
**AAP**	• AAP Policy statement, with 2011 recommendations [10] • Newborn screening for CCHD. Recommendations and resources [3]
**RCPCH**	• Congenital heart disease (Section in the book chapter of Physical examination) [5]
**UK NSC**	• 2014 recommendations [14] • Consultation on the use of pulse oximetry as an additional test in the Newborn and Infant Physical Exam (Cover note) [15]
**Cochrane Library**	• Plana 2018 – Pulse oximetry screening for critical congenital heart defects (Systematic review) [9]
**Others**	• Evans 2016 – Newborn Pulse Oximetry Screening Pilot. End Project Report [16] • Kemper 2011 – Strategies for implementing screening for critical congenital heart disease [11] • Martin 2013 – Implementing recommended screening for critical congenital heart disease [17]

*Abbreviations*: *AAP* American Academy of Pediatrics, *AEP* Spanish Association of Paediatrics (Asociación Española de Pediatría), *CDC* Centers for Disease Control and Prevention, *NICE* National Institute for Health and Care Excellence, *PrevInfad* PrevInfad workgroup from the Spanish Association of Primary Care Pediatrics, *RCPCH* Royal College of Paediatrics and Child Health, *UK NSC* UK National Screening Committee, *USPSTF* US Preventive Services Task Force, *WHO* World Health Organization

## Existing recommendations

We summarized the existing recommendations and the strength of recommendations as per their authors in Table 2.

**Table 2:** Summary of existing recommendations

**Source**	**Ref**	**Date**	**General recommendations for newborn pulse oximetry screening for CCHDs**
**AEP**	[6]	2018	“There is sufficient evidence to recommend neonatal screening by pulse oximetry in the first hours post birth, in addition to prenatal ultrasound and the physical examination.” (Level of evidence A) “The timing of screening affects its sensitivity, with a higher sensitivity the earlier it is performed.” (Level of evidence A) “Early screening, within 24h of birth, reduces the risk of onset with severe or very severe symptoms in CCHD at the expense of a greater number of false positives, although most of the latter are indicative of other disorders that may also require observation, diagnosis and treatment, so early screening is preferable to late screening (>24 h). Very early screening (<12 h) may result in an excessive number of false positives, an issue that needs to be weighed at the local level. In case of very early discharge, screening should be performed before discharge, regardless of timing. It is recommended that the screen be performed between 6 and 24 h post birth.” (Level of evidence B)
**CDC**	[4]	2018	The CDC recommends screening of all newborns in well-baby nursery at ≥24 hours of age or shortly before discharge if <24 hours of age, with a subsequent algorithm according to findings (reported by Kemper et al. [11]). In addition, it is recommended that ‘Pulse oximetry screening should not replace taking a complete family health history and pregnancy history or completing a physical examination, which sometimes can detect a critical CHD before the development of low levels of oxygen (hypoxemia) in the blood.’
**NICE**		2015	“heart; check position, heart rate, rhythm and sounds, murmurs and femoral pulse volume”
**AAP**	[3, 10]	2019	• “All newborns at risk for undetected CCHD should be screened. In other words, the only babies who do not need to be screened are those who are already known to have CCHD, such as those identified by prenatal ultrasound or who have already had an echocardiogram.” • “Screening should begin after 24 hours of age or shortly before discharge if the baby is less than 24 hours of age. Waiting until 24 hours of life will decrease the false-positive results.” • “The screening should occur in the right hand and either foot. If using only one pulse oximeter, test one right after the other.” • “CCHD screening should be conducted by individuals who have pulse-oximetry testing within their scope of practice, who are trained in the use of pulse oximetry and the CCHD algorithm, and who regularly use pulse oximetry for other purposes.” • “In the event of a positive screening result, CCHD needs to be excluded with a diagnostic echocardiogram. Infectious and pulmonary causes of hypoxemia should also be excluded.”
**RCPCH**	[5]	2019	“Until the result of this study [using PO in 15 NHS Trusts in England] are available, it [PO] cannot be recommended as a routine addition to the existing newborn physical examination tests within 72 hours of birth.”
**UK NSC**	[14]	2014	• “A systematic population screening programme is not recommended.” • “The UK NSC recommends piloting the use of the pulse oximetry test to evaluate the potential benefits of its use as a new screening test for congenital heart disease.”
	[15]	2019	• “Recommendation against using pulse oximetry as an additional test in the newborn and infant physical exam”

*Abbreviations*: *AAP* American Academy of Pediatrics, *AEP* Spanish Association of Paediatrics (Asociación Española de Pediatría), *CCHD* Critical congenital heart defect, *CDC* Centers for Disease Control and Prevention, *NHS* UK National Health Service, *NICE* National Institute for Health and Care Excellence, *PO* Pulse oximetry, *RCPCH* Royal College of Paediatrics and Child Health, *UK NSC* UK National Screening Committee, *USPSTF* US Preventive Services Task Force

## Existing evidence

### Short history of PO used for newborns screening

In 2009, the AAP and the American Heart Association (AHA) published conjointly a statement of evidence on the routine use of PO in newborns for detection of CCHDs [1]. The evidence supporting this document was mainly based on two studies (de Wahl 2009 and Riede 2010), both included in the Cochrane systematic review by Plana et al. Although the document showed benefits of PO screening, it was concluded that “future studies in larger populations and across a broad range of newborn delivery systems are needed to determine whether this practice should become standard of care in the routine assessment of the neonate”. Based on this document, the Secretary’s Advisory Committee on Heritable Disorders in Newborns and Children (SACHDNC) recommended that CCHDs to be added to the recommended uniform screening panel (RUSP). After this, the SACHDNC in collaboration with the AAP, the AHA, and the American College of Cardiology Foundation convened a work group to elucidate how to implement this screening safely and with efficiency. The working group evaluated the new findings of large population-based screening activities in Sweden and England, and published their updated recommendations in 2011, which are the basis for the AAP and CDC current recommendations [11]. Previously called “cyanotic congenital heart diseases”, this group recommended renaming the target conditions of the PO screening as “critical congenital heart disease” (CCHD), as “many newborns with the targeted congenital heart defects do not develop clinically appreciable cyanosis until after nursery discharge, and some lesions (e.g., hypoplastic left heart syndrome) may present with significant cardiovascular compromise without apparent cyanosis.” As a conclusion, “the work-group members found sufficient evidence to begin screening for low blood oxygen saturation through the use of pulse-oximetry monitoring to detect CCHD in well-infant and intermediate care nurseries”, however they identified research gaps, mainly regarding some specifics populations and delivery strategies. Therefore, “the Secretary of the US Health and Human Services has directed an interagency work group to develop a plan to address these critical gaps before recommending that CCHD be a part of the recommended uniform screening panel” [11]. The CCHD newborn screening was added to the RUSP in 2011, and two years later, another document was published, addressing some of the implementation challenges [17].

While routine PO screening for detection of CCHDs in all newborns has been introduced in the US, other countries have been conducting similar assessments to evaluate the potential harms and benefits of introducing the screening at a national level. In the UK, a pilot study was conducted in 2015 in order to elucidate effectiveness and feasibility of the implementation of the CCHD newborn screening (findings below). In addition, “Public Health England undertook a review of the extent to which PO met the UK NSC criteria for screening, particularly focussing on the harms and benefits of potential for over-diagnosis, over-treatment, false-positives, false reassurance, uncertain findings, and complications” [15]. Based on the pilot study and the review conclusions, the UK NSC recently recommended against the introduction of routine PO screening, which is disputed by others [18]. In May 2019, the UK NSC “announced a public consultation on its decision not to introduce routine PO for CCHD in all newborn babies” [15]. By the time this review got published, this public consultation revealed that despite the national recommendation against newborn PO screening, 96/189 (51%) neonatal units were currently using it [19]. Of them, 75 (78%) neonatal units felt that screening did not increase unnecessary investigations, and 10 (10%) felt “that any small increase was justified and offset by the benefits of identifying considerable cardiac and non-cardiac pathology” [19]. Findings of this survey call for unified national recommendation.

### Accuracy of PO for detection of CCHDs

#### Threshold for considering PO result pass or failed

As mentioned by Kemper et al, “selecting the threshold for a positive pulse-oximetry monitoring result is challenging, because it must trade-off the harm of missing CCHD against the harm of false-positive screen results” [11]. Several institutions currently agree on the following criteria to consider a screen failed: any oxygen saturation measure less than 90% in the initial screen or in repeated screens; oxygen saturation less than 95% in the right hand and foot on three measures, each separated by one hour; or an absolute difference in oxygen saturation of three percentage points between the right hand and foot on three measures, each separated by one hour [3, 4, 6, 11, 20].

#### Studies reporting accuracy of PO for detection of CCHDs

Accuracy of PO for detection of CCHDs was assessed by one Cochrane review [9], and by one project conducted in the UK [16]. The Cochrane review conducted by Plana et al assessed the diagnostic accuracy of screening with PO compared to echocardiography or clinical follow-up in the first 28 days of life for detection of CCHD in asymptomatic newborn infants [9]. The literature search was conducted up to March 2017. Twenty-one studies were included (16 prospective cohorts and five retrospective cohorts), published between 2002 and 2017, mainly from Europe (UK, 5 studies; Italy, 2 studies; Germany, Norway, Poland, Sweden, Switzerland, and Turkey, 1 study each), but also from other settings (US, 3 studies; Australia, China, Mexico, Saudi Arabia, and South Africa 1 study each). Across the included studies, data was provided for 457,202 newborns and several pulse oximeters models were used. Different thresholds were used to establish a pass or failed screening: post-ductal saturation <95% (8 studies), post-ductal saturation ≤95% (3 studies), pre- and post-ductal saturations <95% (6 studies), pre- and post-ductal saturations ≤94% (1 study), post-ductal saturation <96% (1 study). The applicability concerns were judged as low for all included studies per the review authors.

In 2013, the UK NSC decided to assess the feasibility and impact of PO screening in a wide context, and started a multicentric pilot study across England [16]. This took place from July to December 2015, and it consisted in taking pre- and post-ductal measurements between 4 and 8 hours of life.

#### Main findings

Plana et al considered <95% or ≤95% as a threshold to include studies for primary analysis [9]. From 19 studies (*n* = 436,758), they found that PO for detection of CCHDs had a sensitivity of 76.3% (95% confidence interval [CI] 69.5 to 82.0%) and a specificity of 99.9% (95% CI 99.7 to 99.9%) with a false-positive rate of 0.14% (95% CI 0.07 to 0.22) [9]. Summary positive and negative likelihood ratios were estimated at 535.6 (95% CI 280.3 to 1023.4) and 0.24 (95% CI 0.18 to 0.31), respectively. As detailed by the review authors and considering a median prevalence of 0.6 per 1000 newborns, ‘these results showed that out of 10,000 apparently healthy late preterm or full-term newborn infants, six will have CCHD’; ‘screening by PO will detect five of these infants as having CCHD and will miss one case’ and ‘screening by PO will falsely identify another 14 infants out of the 10,000 as having suspected CCHD when they do not have it’ [9]. The certainty of the evidence was graded as high for specificity and low for sensitivity, downgraded due to serious imprecision (due to small number of cases with CCHD included) and serious risk of differential verification bias (‘diagnosis was established by echocardiography in test positive cases however test negatives were usually confirmed by clinical follow-up or by accessing congenital malformation registries and mortality databases’) [9].

Main findings from the English pilot study showed that among the 32,836 newborns screened, there were 239 cases (0.73%) with a positive screen, out of which eight were CCHD cases [16]. The screening was performed within the target time of 4 to 8 hours for half (52%) of the participants, within 12 hours for 78%, and after 24 hours for 8.5%. More in detail, around half the newborns with a positive screen were admitted to the neonatal unit. Among those not admitted, 97% had transitional circulation and 2 cases had culture negative sepsis. Among the 114 newborns admitted to the neonatal unit, eight (7%) had a CCHD, 86 (75%) had a ‘significant illness which required medical intervention’, and 22 (9% of newborns with positive screen, 0.07% of all newborns screened) were healthy babies. From these findings, investigators interpreted that ‘earlier screening (within 24 hours) results in a higher proportion of babies detected with a clinical condition but at the expense of a slightly higher screen positive rate’. Two CCHD cases were missed by a negative screen result. These two newborns also passed the antenatal ultrasonography screening and the clinical examination screening without detecting anomalies. One of them had a fatal outcome, and the other one presented with cardiovascular collapse. Overall, authors concluded that ‘the pilot has demonstrated that in general, it is feasible to introduce PO screening in an NHS environment, however there are important clinical considerations’ and that ‘the routine introduction of PO screening could be considered once these issues have been satisfactorily resolved.’

#### Accuracy considering different thresholds

Different thresholds were considered by two studies included in the Cochrane review [9]. According to the review authors, one study used a threshold of ≤94% and found a sensitivity of 100% (95% CI 29 to 100) and specificity of 100% (95% CI 100 to 100); and another study used a threshold of <96%, and found a sensitivity of 100% (95% CI 3 to 100) and specificity of 100% (95% CI 100 to 100), respectively [9]. While it is difficult to understand 100% for both sensitivity and specificity, we noted some inconsistencies between the findings of this systematic review on this aspect and what is reported in the original included papers. We have contacted the review authors for clarification but have not received any reply.

#### Accuracy considering antenatal diagnosis

The review authors looked whether antenatal diagnosis could have an impact in the findings and found that both sensitivity and specificity did not change significantly when newborns with suspicion of CHD by antenatal ultrasound screening were included versus excluded [9].

#### Accuracy considering risk of bias

The Cochrane review authors also looked at the impact of risk of bias in the findings [9]. For the ‘flow and timing domain’, nine studies were judged at unclear risk of bias and 10 studies at low risk of bias. It was found that risk of bias for this domain had no significant effect on sensitivity, but that studies classified at unclear risk of bias had higher specificity than those judged at low risk of bias (100% [95% CI 99.9 to 100%] versus 99.7% [95% CI 99.3 to 99.8%]; *P* = 0.016).

#### Accuracy considering other factors

Other factors that could impact the findings of pulse-oximetry in the detection of CCHDs have been reported. Algorithm and cut-offs for pass or failed PO screening were based on studies conducted at low altitude. As oxygen saturations are lower at higher altitudes, using the same algorithm in newborns at high altitudes might lead to higher false-positive rate. While there is global awareness around this question, there is a lack of data to support modification of the current algorithm on PO screening [3, 11]. The altitude where screening was performed was not reported in the studies included in the Cochrane review [9].

Performing the screening in alert newborns was associated with lower false-positive rates, ‘possibly by reducing the likelihood of low oxygen saturations caused by hypoventilation in deep sleep’, although this was reported from anecdotal reports [11]. Performing PO screening around the time of the hearing screening was also associated with an improved efficiency, ‘assuming that the hearing screening is conducted after 24 hours or immediately before discharge’ [11].

#### Conclusions on accuracy of PF for detection of CCHDs

Current evidence supports consistent accuracy for detection of CCHDs in newborns by pulse oximetry screening in addition to antenatal ultrasonography and clinical examination.

### Timing of PO screening

With the closure of the arterial ductus and other physiologic changes after birth, timing might have an impact in the findings of PO screening. A recent study estimated the median ductal closure time to be 27 hours in boys and 45 hours in girls [5]. However, the cut-off considered in the studies is usually 24 hours, which coincides with timing discharge of apparently healthy neonates without maternal complications leading to performing neonatal examination and screening within the first 24 hours of life. In the review conducted by Plana et al (primary analysis), PO screening was performed within the first 24 hours of life in eight studies, with an overall sensitivity of 79.5% (95% CI 70.0 to 86.6) and specificity of 99.6% (95% CI 99.1 to 99.8) [9]. Among the 11 studies that performed the screening test after 24 hours of life, the overall sensitivity was 73.6% (95% CI 62.8 to 82.1) and specificity 99.9% (95% CI 99.9 to 100). There were no significant differences on sensitivity between performing the screening before versus after 24 hours of life, but the false-positive rate for detection of CCHDs was significantly lower among the group of newborns screened after 24 hours of life (0.06% [95% CI 0.03 to 0.13] versus 0.42% [95% CI 0.20 to 0.89]; *P* = 0.027).

The Spanish National Neonatal Society affirmed the opposite: “The timing of screening affects its sensitivity, with a higher sensitivity the earlier it is performed” with a level of evidence judged as A. From the supporting evidence provided by the consensus document, authors argued that “an analysis of late screens (>24 hours) demonstrated that half of CCHDs manifest in the first 24 hours and 20% do so with cardiovascular compromise”, but no data was given on direct comparison between screening before versus after 24 hours of life. However, there is a general agreement in the literature that early screening leads to an increased rate of false-positive cases, although it is highly valuable to detect most of these cases, as most of them are indicative of other severe disorders that may also require prompt diagnosis and treatment. Indeed, in the UK, early screening of around 26,000 newborns led to a screening positive rate of 0.8%, with the detection of nine CCHDs cases and within the false-positive cases, 79% had a significant medical condition [21]. Conversely in the US, late screening of around 73,000 newborns led to a very low false-positive rate of 0.04%, at the expenses of a low number of CCHDs cases detected (three cases). As reflected by Ewers et al, “the likelihood is that in the US cohort, many infants with CCHD presented before screening took place” [21]. These considerations are the main rationale why early screening is finally recommended by several associations, such as the Spanish National Neonatal Society, as it is judged that benefits outweigh risks.

#### Conclusions on timing of oximetry

- The timing of pulse oximetry screening had no significant difference on sensitivity, but the false positivity rate was significantly lower among newborns screened after 24 hours of life, when compared with newborns screened within the first 24 hours of life.
- There is some evidence – and wide acceptance among physicians – that early screening leading to early detection of CCHDs allows timeliness of appropriate medical intervention and therefore reduces the risk of onset with severe or very severe symptoms, with subsequent better outcome.
- Newborns with false-positive screening results might undergo unnecessary additional tests such as echocardiogram. However, early screening allows detection of hypoxemia due to other clinically severe conditions that benefit from prompt intervention.
- Overall, benefits of early screening are judged to be greater than the potential negative impact of the increased rate of positive rate by some societies, but this is not the position of other institutions such as the United Kingdom National Screening Committee and the Royal College of Paediatrics and Child Health.

### Post-ductal versus pre- and post-ductal screening

A pulse oximeter device can measure pre-ductal oxygen saturation when the probe is placed on the right hand, and post-ductal oxygen saturation when used on either foot. Eleven studies included in the Plana et al (primary analysis) review measured post-ductal oxygen saturation only for the detection of CCHDs, with a sensitivity of 81.2% (95% CI 70.9 to 88.4), a specificity of 99.9% (95% CI 99.7 to 100), and a false-positive rate of 0.13% (95% CI: 0.05 to 0.31%) [9]. Pre-ductal and post-ductal screening was performed in 8 studies, with a sensitivity of 71.2% (95% CI 58.5 to 81.3), a specificity of 99.8% (95% CI 99.5 to 99.9), and a false-positive rate of 0.17% (95% CI 0.06 to 0.46). It was found that the site of testing (post-ductal versus pre- and post-ductal) had no significant effect on sensitivity nor specificity.

#### Conclusions on site of testing

The site of testing (post-ductal versus pre- and post-ductal) had no significant effect on sensitivity nor specificity for detection of CCHDs.

### Potential harms of PO screening

PO is a safe and harmless tool. Newborns with a false-positive screen will however receive additional testing such as an echocardiography or a chest radiography, and might be referred or admitted to a neonatal unit. This has the potential to cause discomfort to the newborn, although additional tests are very unlikely to be invasive. Authors from the pilot study conducted in the UK concluded that “there was little evidence of additional significant harm to the majority of babies who had a screen positive outcome. It is possible however, that some babies underwent unnecessary admission and investigation as a result of testing screen positive, particularly some of those with culture-negative sepsis, these are likely to be in a minority” [16]. In settings where echocardiography is not available within a short period, the delay in performing the test for confirmation or exclusion of the CCHDs diagnosis might increase parental anxiety, in addition to increase the workload for health workers performing echocardiography and additional potential admissions until the test is performed. Indeed, like any screening of a potential severe disease, CCHDs screening might raise unnecessary parental anxiety. However, clinical practice and available literature suggests good acceptability to mothers and no increased anxiety among mothers given false-positive results compared to mothers given true-negative results [12].

However, it was found that the number of false-positive results generated by PO screening was lower than those generated by clinical examination alone, which is worth to consider when balancing benefits and harms of PO screening [22].

In addition, it is important to note that detection of hypoxemia that is not due to CCHDs allows the identification of many cases of clinically significant conditions that cause hypoxemia such as pneumonia or sepsis, that benefit from early recognition and management [6, 16]. As neonates with a clear non-cardiac diagnosis are unlikely to require an echocardiography, the number of false-positive cases lead to a lower number of echocardiography needed. In the UK, health staff were not aware of any increase in the number of echocardiograms during the period of the pilot study introducing PO screening [16].

#### Conclusions on harms and limitations of PO

Overall, early diagnosis of CCHD with pulse oximetry is judged to be beneficial and cost-effective, and potential harms associated with false-positive tests are not serious, while missing CCHDs and other serious diseases detected by hypoxaemia in absence of pulse oximetry screening can lead to serious consequences.

## Summary of findings

- Current evidence supports consistent accuracy for detection of CCHDs in newborns by pulse oximetry screening in addition to antenatal ultrasonography and clinical examination.
- Overall, early diagnosis of CCHD with pulse oximetry is judged to be beneficial and cost-effective, and potential harms associated with false-positive tests are not serious, while missing CCHDs and other serious diseases detected by hypoxaemia in absence of pulse oximetry screening can lead to serious consequences.
- Timing of oximetry:
  - ○ The timing of pulse oximetry screening had no significant difference on sensitivity, but the false positivity rate was significantly lower among newborns screened after 24 hours of life, when compared with newborns screened within the first 24 hours of life.
  - ○ There is some evidence – and wide acceptance among physicians – that early screening leading to early detection of CCHDs allows timeliness of appropriate medical intervention and therefore reduces the risk of onset with severe or very severe symptoms, with subsequent better outcome.
  - ○ Newborns with false-positive screening results might undergo unnecessary additional tests such as echocardiogram. However, early screening allows detection of hypoxemia due to other clinically severe conditions that benefit from prompt intervention.
  - ○ Overall, benefits of early screening are judged to be greater than the potential negative impact of the increased rate of positive rate by some societies, but this is not the position of other institutions such as the United Kingdom National Screening Committee and the Royal College of Paediatrics and Child Health.
- The site of testing (post-ductal versus pre- and post-ductal) had no significant effect on sensitivity nor specificity for detection of CCHDs.

## Data Availability

Not applicable.
